# Cardiac optogenetics: shining light on signaling pathways

**DOI:** 10.1007/s00424-023-02892-y

**Published:** 2023-12-14

**Authors:** Siri Leemann, Franziska Schneider-Warme, Sonja Kleinlogel

**Affiliations:** 1 Institute of Physiology, University of Bern, Bern, Switzerland; 2 Institute for Experimental Cardiovascular Medicine, University Heart Center Freiburg - Bad Krozingen, and Medical Faculty, University of Freiburg, Freiburg, Germany; 3 F. Hoffmann-La Roche, Translational Medicine Neuroscience, Basel, Switzerland

**Keywords:** Cardiac optogenetics, Cardiac signaling, GPCR, OptoGPCR

## Abstract

In the early 2000s, the field of neuroscience experienced a groundbreaking transformation with the advent of optogenetics. This innovative technique harnesses the properties of naturally occurring and genetically engineered rhodopsins to confer light sensitivity upon target cells. The remarkable spatiotemporal precision offered by optogenetics has provided researchers with unprecedented opportunities to dissect cellular physiology, leading to an entirely new level of investigation. Initially revolutionizing neuroscience, optogenetics quickly piqued the interest of the wider scientific community, and optogenetic applications were expanded to cardiovascular research. Over the past decade, researchers have employed various optical tools to observe, regulate, and steer the membrane potential of excitable cells in the heart. Despite these advancements, achieving control over specific signaling pathways within the heart has remained an elusive goal. Here, we review the optogenetic tools suitable to control cardiac signaling pathways with a focus on GPCR signaling, and delineate potential applications for studying these pathways, both in healthy and diseased hearts. By shedding light on these exciting developments, we hope to contribute to the ongoing progress in basic cardiac research to facilitate the discovery of novel therapeutic possibilities for treating cardiovascular pathologies.

## Introduction

Optogenetics uses naturally occurring and genetically engineered rhodopsins to render target cells light-sensitive and command physiological activity with millisecond precision. The high spatiotemporal control of optogenetics has granted researchers power over cellular excitability in a never-before-seen manner. Over the past decade, optogenetic approaches have been established in cardiac electrophysiology research, and various optical tools have been used to monitor, control, and manipulate the membrane potential of cardiac cells. In contrast, optogenetic control over specific signaling events within the heart is still in its infancy. Here, we review the established optogenetic toolbox to investigate and control signaling pathways in the healthy and diseased heart, focusing on G-protein coupled receptor (GPCR) signaling and downstream intracellular signaling pathways.

### Cardiac signaling pathways

The heart is a muscular organ comprising four chambers, rhythmically pumping oxygenated blood to the body’s periphery. Heart tissue consists of cardiac muscle cells (cardiomyocytes), intracardiac neurons, fibroblasts, mural cells, endothelial cells, and immune cells, which are distributed differently between atrial and ventricular walls [1]. Cardiac contractions are coordinated by a precisely timed series of electrical excitation-repolarization events. The sinus node acts as the primary pacemaker, generating depolarizing signals that travel via the atria to the atrioventricular (AV) node, and, after a delay, to the His-Purkinje system of the ventricles. The ability of cardiomyocytes to convert excitatory electrical impulses into cellular contractions is commonly referred to as excitation–contraction coupling (ECC) [2, 3].

#### Ca^2+^-signaling

**Fig. 1:**
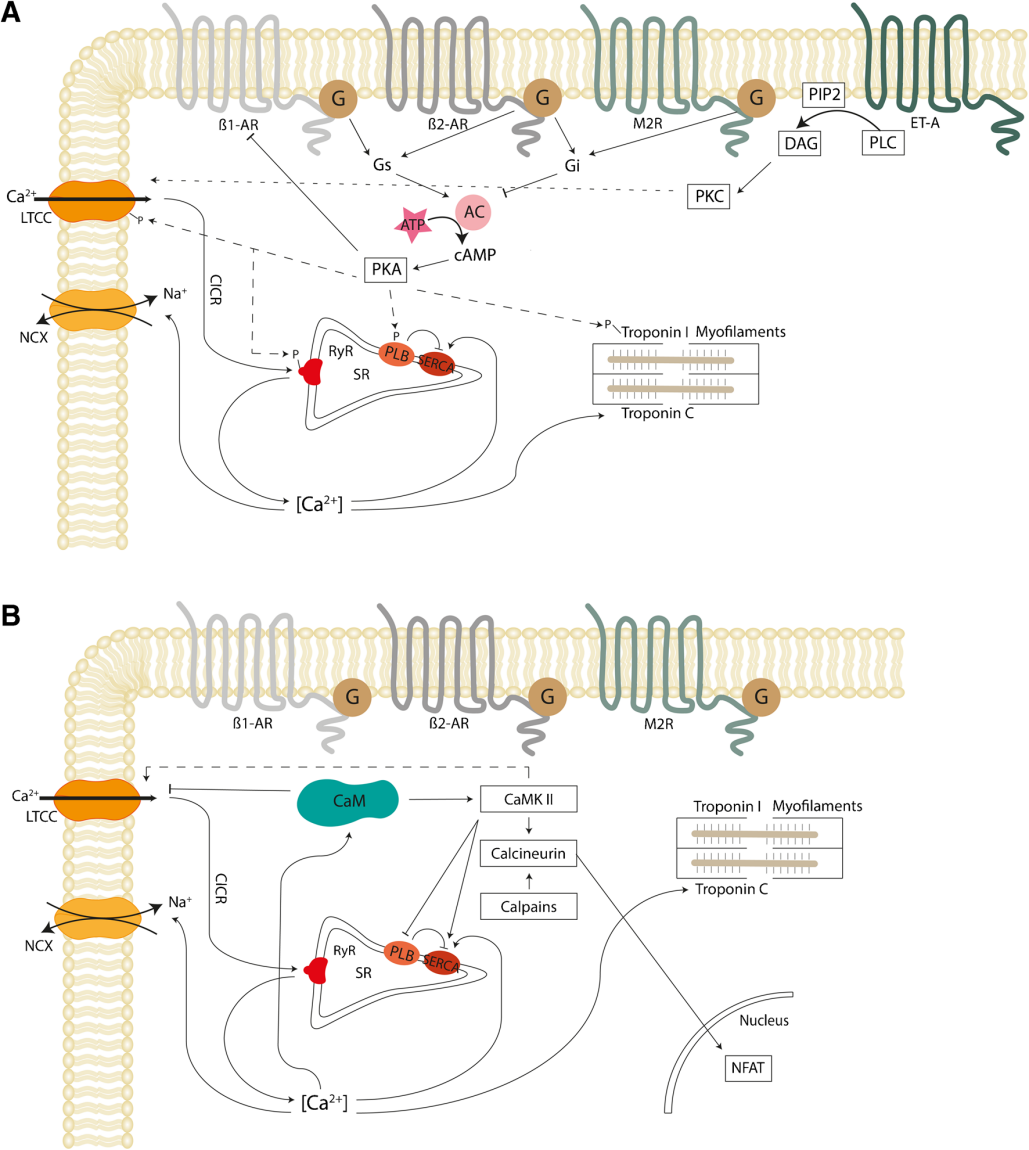
Overview of signaling pathways in cardiomyocytes. **A** Excitation–contraction coupling (ECC) and the effects of GPCR signaling. Ca^2+^ enters via L-type Ca^2+^ channels (LTCC), and activates Ca^2+^ release from the sarcoplasmic reticulum (SR) via the ryanodine receptor (RyR), which leads to an intracellular rise in [Ca^2+^]. Ca^2+^ binds to troponin C and initiates contraction. SR Ca^2+^ re-uptake via the SR Ca^2+^-ATPase (SERCA) and extrusion via the Na^+^/Ca^2+^ exchanger (NCX) lead to Ca^2+^ dissociation from troponin C initiating the relaxation process. Activation of beta-adrenergic receptors (β1-AR, β2-AR) activates adenylate cyclase (AC) to produce cyclic AMP (cAMP) and activates protein kinase A (PKA) via the Gs-signaling pathway. PKA phosphorylates phospholamban (PLB) and regulates SR Ca^2+^ re-uptake, LTCC and RyR activity, resulting in a net increase in Ca^2+^ transient amplitude. Activation of M2-muscarinic receptor (M2R) acts via the Gi-inhibitory pathway resulting in a net decrease of cAMP levels. Activation of endothelin-1 (ET-1) activates phospholipase C (PLC) and consequently protein kinase C (PKC). **B** Important Ca^2+^-handling and Ca^2+^-binding proteins. Following an intracellular [Ca^2+^] increase, calmodulin (CaM) is activated, which in turn activates calmodulin-dependent protein kinase II (CaMKII) and the Ca^2+^-dependent phosphatase calcineurin. Calmodulin has an inhibitory effect on LTCC, whereas CaMKII activates LTCC. Finally, calcineurin stimulates the transcription factor NFAT in the nucleus. ATP, adenosine triphosphate; CICR, calcium-induced calcium release; PIP2, phosphatidylinositol 4,5-bisphosphate; DAG, diacylglycerol

The ECC process requires meticulous regulation. Ca^2+^ is the key regulator of cardiomyocyte contractility and plays major roles in controlling mitochondrial bioenergetics and cell death. When the membrane potential reaches the activation threshold of -70 mV in working cardiomyocytes, a rapid influx of Na^+^ via fast voltage-gated Na^+^ channels initiates the cardiac action potential. Depolarization beyond 0 mV causes the opening of voltage-gated Ca^2+^ channels (mainly L-type Ca^2+^ channels (LTCC)), leading to an influx of Ca^2+^ into cardiomyocytes. The intracellular rise in Ca^2+^ triggers the release of Ca^2+^ from the sarcoplasmic reticulum (SR) via ryanodine receptors (RyR), a process called calcium-induced calcium release (CICR). Intracellular Ca^2+^ binds to troponin C at the sarcomere, promoting myocyte contraction by exposing the myosin-binding site, allowing actin-myosin interaction. Elevated intracellular Ca^2+^ is rapidly cleared by the sarcoplasmic/endoplasmic Ca^2+^-ATPase (SERCA) and via the Na^+^/Ca^2+^ exchanger (NCX) at the sarcolemma, exporting Ca^2+^ when acting in forward mode (Fig. 1) [2, 3].

Functioning as one of the central second messengers, Ca^2+^ binds to several Ca^2+^-binding proteins, including calmodulin, in turn modulating the activity of calmodulin-dependent protein kinase II (CaMKII), calcineurin, and calpains (Fig. 1B). When calmodulin is activated, it undergoes a conformational change exposing protein interaction sites essential to activate CaMKII, a serine/threonine kinase, and calcineurin, a serine/threonine phosphatase [4–7]. Accordingly, calmodulin is activated by elevated intracellular Ca^2+^ levels, leading to an activation of CaMKII, which results in phosphorylation of LTCC increasing the open probability of these channels. Additionally, Ca^2+^-dependent inactivation of LTCC is also mediated by calmodulin. Calmodulin is pre-bound to the C-terminal region of the α-subunit of LTCC during diastole. When the Ca^2+^ concentration reaches micromolar levels upon depolarization, Ca^2+^ binds to the C-terminus of calmodulin, which then interacts with the C-terminal region of LTCC to induce channel inhibition [5, 6]. Additionally, CaMKII influences RyR activation during ECC by enhancing Ca^2+^ release from the SR. CaMKII also phosphorylates SERCA and phospholamban (PLB), thereby regulating Ca^2+^-reuptake into the SR [6]. Beyond these effects, CaMKII directly modulates contractility via phosphorylation of the myofilaments. Interestingly, Ca^2+^ levels also regulate the β-adrenoceptor (AR) signaling pathway through inhibition of adenylyl cyclase (AC). A Ca^2+^-dependent decrease in cAMP levels is further promoted by calmodulin-mediated activation of phosphodiesterases [8] (Fig. 1B). Finally, calpain, which is activated at high Ca^2+^ concentrations, has been described to cleave proteins in pathological settings, such as during Ca^2+^ overload following ischemia–reperfusion and in heart failure conditions [9]. ECC is closely regulated by the autonomic and the neuroendocrine systems adjusting heart function to the daily changing systemic demands, such as during exercise or sleep [10].

#### Autonomous nervous system and neuro-humoral signaling pathways

The autonomic nervous system is comprised of two major antagonistic branches, the sympathetic and the parasympathetic systems [11]. Sympathetic stimulation leads to an increase in contractility (ionotropy), heart rate (chronotropy), speed of relaxation (lusitropy), and conduction velocity at the AV node (dromotropy). It exerts its effects through the release of norepinephrine, which binds to AR expressed in the sinoatrial and AV nodes, as well as on atrial and ventricular cardiomyocytes [12] (Fig. 1A). While the β1-AR accounts for the largest portion of AR in the heart, the β2-AR is also expressed in healthy cardiac tissue [13]. Additionally, the β3-AR, which is only found at very low levels in cardiomyocytes of the healthy heart [14], has been shown to be upregulated in ventricular cardiomyocytes during pathophysiological remodeling [15]. While its upregulation may be a beneficial compensatory mechanism, the precise role of the β3-AR in cardiovascular disease is a topic of broad debate [16]. The β-AR belong to the large family of GPCR. Activated β-AR trigger primarily the Gs (stimulatory) signaling pathway, but have also been shown to stimulate the Gi (inhibitory) pathway, especially during heart failure. Upon activation of the β-AR, the Gs-α subunit dissociates from the heterotrimeric G-protein complex and activates AC, leading to an increase in intracellular cAMP levels, which in turn activates protein kinase A (PKA). Activated PKA acts on multiple pathways and promotes phosphorylation of LTCC and RyR. In working cardiomyocytes, this leads to an increased intracellular Ca^2+^ release, increased excitability, and stronger contractions. Lusitropy is further achieved through phosphorylation-inactivation of PLB. PLB itself is responsible for inhibiting SERCA, so its inactivation results in faster Ca^2+^ reuptake into the SR via SERCA. Additionally, PKA also modifies contractility and relaxation through the direct phosphorylation of troponin I and myofilaments. Finally, PKA also phosphorylates the β-AR, leading to GPCR uncoupling and desensitization (negative feedback loop, Fig. 1A) [10, 17, 18]. In cardiac pacemaker cells, the cAMP-mediated transient increase of intracellular Ca^2+^ concentration together with direct modulation of the funny current (I_f_) increases the velocity of diastolic depolarization and thus their firing rate, resulting in higher heart rate (positive chronotropic effect) [19, 20]. Finally, sympathetic stimulation at the AV node increases the rate of junctional rhythm, thereby speeding-up conduction from the atria to the ventricles [21]. By contrast, postganglionic vagal stimulation of this region leads to a decrease in ventricular rate [22].

In addition to these classical signaling pathways, the β3-AR is also responsible for nitric oxide stimulation [15, 23, 24]. Nitric oxide has a direct effect on ECC: when nitric oxide concentration is low, nitrosylation of the LTCC and the RyR lead to increased intracellular Ca^2+^ concentration and additional CICR. On the contrary, when nitric oxide concentration is high, the guanylate cyclase is activated, which in turn leads to the synthesis of cyclic guanosine monophosphate (cGMP). cGMP then stimulates phosphodiesterase II, which is responsible for cAMP degradation resulting in interruption of the cAMP-PKA pathway [25].

GPCR also regulate the parasympathetic branch of the autonomic nervous system. Acetylcholine released from parasympathetic neurons activates muscarinic receptors, especially M2-receptors, expressed in nodal cells, atrial tissue, and, to a lower extent, in the ventricles [26]. Muscarinic M2-receptors primarily couple to G-proteins. When Gi dissociates from the G protein complex, it inactivates AC, thereby decreasing intracellular Ca^2+^ levels by inhibiting the cAMP-PKA axis (Fig. 1A). Additionally, the Gi-βγ subunits open G-protein-gated inward rectifying potassium channels (GIRK channels, mediating I_K,ACh_), inducing membrane hyperpolarization and action potential shortening of atrial cardiomyocytes (Fig. 1A) [26, 27].

During heart failure, an overactive sympathetic nervous system serves as a compensatory mechanism to sustain cardiac performance and to enhance contractility. However, prolonged stimulation of the sympathetic nervous system can reduce cardiac contractility and contractile strength (inotropic reserve). At the molecular level, heart failure is marked by myocardial β-AR dysfunction, which involves a significant reduction (approximately 50%) in β1-AR density at the cell membrane (downregulation) and the detachment (desensitization) of β1-AR and β2-AR from G proteins [16]. Additionally, in cardiac disease, the expression of β3-AR is increased, and they exhibit reduced desensitization compared to β1-AR and β2-AR. Finally, it has been established that endothelin-1 (ET-1) is implicated in cardiac hypertrophy and, ultimately, in heart failure, mainly due to its function as a growth factor in a variety of cells, such as vascular smooth muscle cells, cardiac myocytes, and fibroblasts [28].

Important signaling molecules of the neuro-humoral signaling axis are ET-1, angiotensin II (Ang II), and atrial and B-type natriuretic peptides. ET-1 regulates vascular responses and cardiac contractility and plays a role in the development of hypertrophy [29]. ET-1 acts through ET-1 receptors A and B (ET-A and ET-B), which couple to the Gq/11 proteins. Upon activation of the Gq-coupled ET-A and the subsequent stimulation of protein kinase C, the activity of the LTCC and Na^+^/Ca^2+^ exchanger (NCX) is increased, leading to an increase in transmembrane Ca^2+^ influx [30]. Therefore, ET-1 activation modulates components of the excitation–contraction machinery, resulting in a positive inotropic effect similar to that following sympathetic stimulation. Both ET-A and ET-B are expressed in fibroblasts, while the ET-A receptor is the prevalent type in cardiomyocytes [31]. Endothelial cells and myocytes secrete ET-1 upon Ang II activation, for instance in response to mechanical stimuli [32, 33]. Ang II belongs to the renin–angiotensin–aldosterone system, which governs blood pressure by regulating salt and water homeostasis. Chronic stimulation of the renin–angiotensin–aldosterone system can lead to cardiac hypertrophy and remodeling. Ang II receptors (AT-1 and AT-2) also couple to the Gq/11-signaling pathway, increasing cytosolic Ca^2+^ levels upon activation. AT-1 activation can stimulate a number of intracellular signaling cascades, including pathways involving protein kinase C, reactive oxygen species, and tyrosine kinases, and G-protein–independent signaling pathways, such as the MAPK and the Akt/PKB/eNOS pathways.

In summary, Ca^2+^ homeostasis and GPCR signaling are key regulators of cardiomyocyte function and cardiac pathology and therefore make excellent targets to investigate or modulate cardiac signaling.

## Optogenetics

Pioneering efforts led by Oesterhelt in the 1970s resulted in the discovery of bacteriorhodopsin, a light-driven proton pump from the halophilic bacterium *Halobacterium salinarum*, followed by the publication of its amino acid sequence by Khorana and colleagues [34, 35]. In seminal papers published in 2002 and 2003, Nagel and colleagues revealed the function of related microbial rhodopsins from the green algae *Chlamydomonas reinhardtii*, acting as light-gated ion channels, referred to as channelrhodopsins (ChR) [36, 37]. Cation non-selective variants such as *Chlamydomonas* channelrhodopsin-2 (ChR2) mediate depolarizing ion currents upon blue light application [37]. In groundbreaking optogenetic experiments, ChR2 was thus applied to optically trigger action potentials in excitable cells, *i.e.*, in neurons and cardiomyocytes [38, 39].

Optogenetic experiments build on three main pillars: (a) a light-sensitive moiety, usually derived from a natural photoreceptor protein; (b) methods to genetically introduce the light-sensitive moiety into specific cell populations; and (c) techniques to guide precisely timed light pulses to the target tissues, cells, or cellular compartments. While the underlying disciplines each have their own longstanding history, their combination opened the path to the so-called *optogenetic revolution* in biomedical research [40, 41]. With the growing variety of application areas, the interest in developing a larger and more versatile optogenetic toolbox has grown exponentially. This includes both optogenetic actuators for modulating cellular behavior with light, as well as optogenetic sensor proteins for visualizing cell-specific functions within complex biological tissues.

### Cardiac optogenetics

The combination of optogenetic actuators and sensors has launched cardiovascular research into a new era, particularly in terms of understanding and controlling arrhythmias. To date, no other method to study cardiac tissue properties, be it electrical, mechanical, or pharmacological, has matched the spatiotemporal precision and cellular specificity of optogenetics [39, 42–44]. Optical tools have been used in basic cardiac research since the early 1970s, when imaging membrane voltage dynamics with fluorescent dyes, so-called optical mapping, was introduced to study cardiac conduction pathways and arrhythmia mechanisms [45]. Classically, Ca^2+^ concentrations were measured using synthetic fluorescent chelators [46] or recombinant aequorin [47]. While the chelators cannot be targeted to specific cell types, aequorin enables targeting but requires the addition of coelenterazine. Additionally, aequorin signals have low intensity as luminescence produces maximally one photon per co-factor molecule [48]. The development of genetically encoded calcium indicators (GECI) and genetically encoded voltage indicators (GEVI) for optogenetic sensing now opens further opportunities for visualizing cardiac activity within specific cell populations [49, 50]. In contrast to the dyes, GECI allow for cell-specific monitoring of Ca^2+^ dynamics over extended time windows. Moreover, subcellular targeting of GECI has provided insight into subcellular Ca^2+^-signaling mechanisms, such as nuclear, mitochondrial, and SR signaling. For instance, the Ca^2+^ indicator GECO was applied to show that nuclear Ca^2+^ transients were elicited by both electrical and receptor stimulations in ventricular myocytes and that these nuclear Ca^2+^ transients are slower than cytoplasmic Ca^2+^ transients [51]. Shang and colleagues fused the native Ca^2+^ sensor GCamP6f to triadin 1 or junctin, creating a junction-targeted GECI [52]. This novel sensor permits visualization and measurement of nanodomain junctional Ca^2+^ dynamics, important for RyR gating during ECC [53, 54].

The rate of progress in developing optimized GEVI for cardiac applications has lagged behind GECI development, due to difficulties in achieving adequate kinetics and sensitivity. Voltage-sensitive dyes are fast, highly sensitive, and can be spectrally tuned; however, they often suffer from phototoxicity and bleaching. To overcome the limitations in speed and sensitivity of existing GEVI and because of the spectral overlap between these GEVI and optogenetic actuators, near-infrared (NIR) sensors, such as QuasAr [55] or Voltron635, were developed, the latter representing a chemogenetic voltage indicator combining a voltage-sensitive microbial rhodopsin domain with a dye-capturing protein domain [56]. The QuasAr (“quality superior to Arch”) indicators are mutants of archaerhodopsin 3 (Arch), which function as fast and sensitive voltage indicators with the furthest red-shifted spectrum of all GEVI, thereby offering the unique capability of cross-talk-free all-optical electrophysiology in combination with most optogenetic actuators [57].

The introduction of GECI and GEVI happened around the same time the first optogenetic actuators (ChR2, halorhodopsin) were applied in neuroscience [58]. Suddenly, it seemed achievable to create all-optical, closed-loop control systems by combining optogenetic actuators with sensors for control of function with an intrinsic feedback mechanism. Early work by Miesenböck and colleagues already highlighted the benefits of all-optical closed-loop optogenetics, in which light is used to exert control over and simultaneously sense biological processes [59–61]. Such closed-loop optical systems have been broadly utilized, especially within the field of neuroscience, and are discussed in comprehensive reviews [62, 63]. In the cardiac field, all-optical approaches additionally provided a unique opportunity to facilitate the lengthy and costly drug development pipeline. For instance, preclinical testing for drugs involves a cardiotoxicity assay, which is currently based on compound testing in cell systems, for example testing effects on voltage-gated K^+^ channel subfamily H member 2 (hERG) channels, a main off-target for drugs. Cohen and colleagues combined QuasAr with a blue light-gated ChR variant creating a macroscopic platform called Optopatch™, used for cardiotoxicity testing in stem cell–derived cardiomyocytes [55, 64]. Similarly, Entcheva and colleagues developed a platform, called OptoDyCE, for all-optical, dynamic cardiac electrophysiology. OptoDyCE combines ChR2 with spectrally compatible synthetic dyes or optogenetic sensors [65]. Streit and colleagues employed similar “tandem” proteins to create the first light-induced, bi-directional electrophysiology platform (LiEp) enabling powerful, high-throughput, and affordable drug screening of voltage-gated ion channels, such as hK_v_1.5, hNa_v_1.5, and hERG [57]. Finally, an all-optical, closed-loop platform was applied to monitor and control electrical activity by restoring normal electrical activity after AV block and manipulating the propagation of the electrical wavefront [66]. These platforms will likely influence drug discovery and development in the future, as some of them have already been translated to industry.

#### Use of microbial ion pumps and light-gated ion channels for membrane potential modulation in cardiac cells

The classical optogenetic actuator toolkit is based on microbial rhodopsins and includes excitatory, depolarizing ion channels, such as ChR2, and inhibitory, hyperpolarizing ion pumps, such as halorhodopsin (NpHR) from the archaebacterium *Natromonas pharaonic* [67, 68] or the chloride pump ArchT [69]. These rhodopsins possess the unique ability to modulate the membrane potential upon illumination. This has been widely used to either elicit or suppress action potentials in excitable cells, such as neurons and cardiomyocytes.

**Fig. 2:**
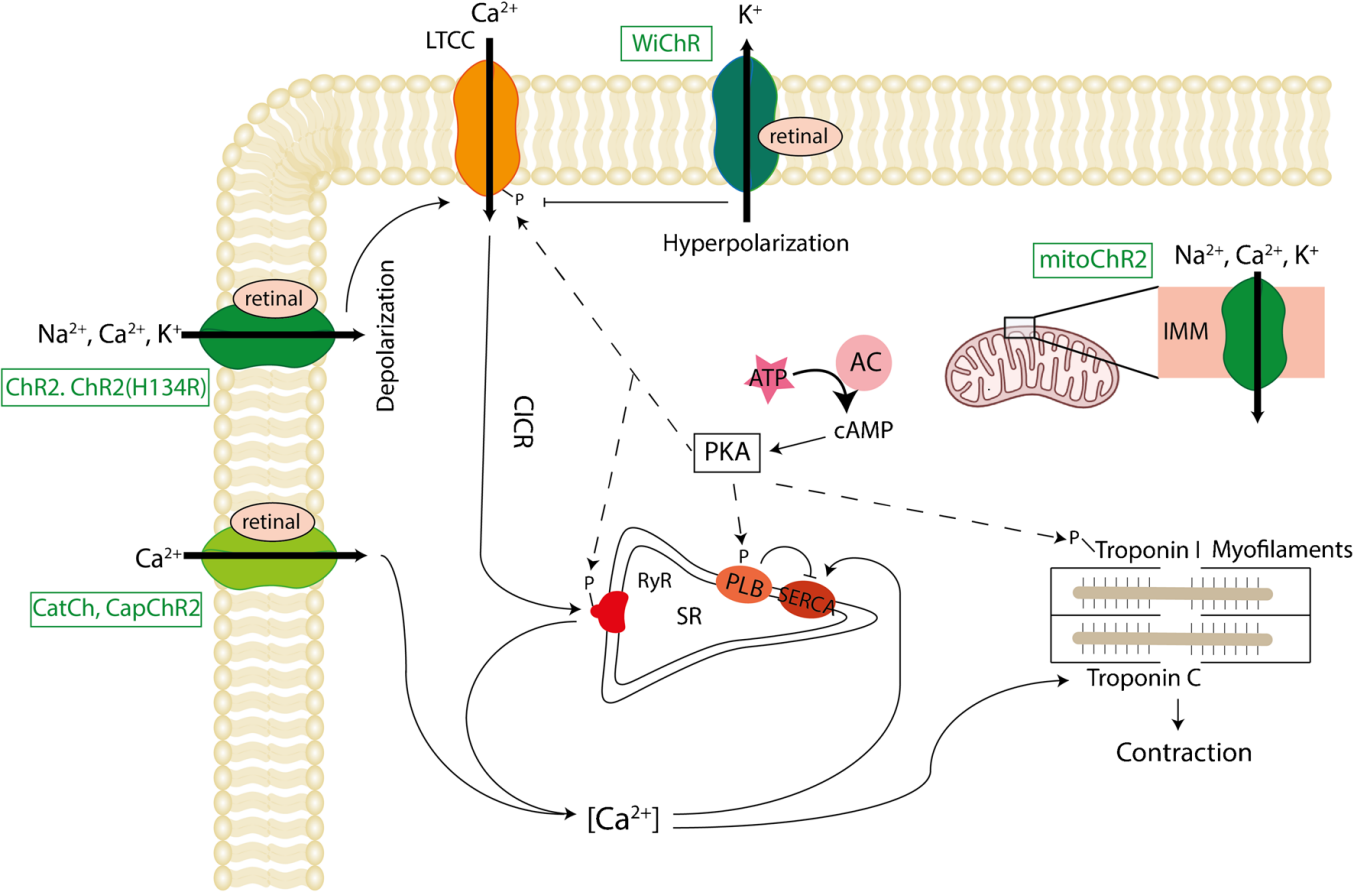
Microbial opsin-based tools applied in cardiomyocytes. Channelrhodopsin (ChR2)-based tools and their effects are depicted. ChR2 has been expressed in the sarcolemma and in the inner mitochondrial membrane. ChR2, ChR2(H134R), CatCh, CapChR2 lead to depolarization, while WiChR is an optogenetic inhibitor. The mitochondrial-targeted ChR2, mitoChR2, also leads to an influx of ions into the mitochondria. AC, adenylyl cyclase; ATP, adenosine triphosphate; cAMP, cyclic AMP; CICR, calcium-induced calcium release; IMM, inner mitochondrial membrane; PKA, protein kinase A; PLB, phospholamban; RyR, ryanodine receptor; SERCA, SR Ca^2+^-ATPase; SR, sarcoplasmatic reticulum

To this day, ChR2, more specifically the H134R point mutant [58], which shows increased Na^+^ conductivity and improved retinal binding in some model systems, is the most widely used optogenetic actuator in the heart. In 2010, Brügmann et al. demonstrated that blue light pulses could be used to optogenetically pace cardiomyocytes and to terminate arrhythmias [39]. Ca^2+^ imaging showed that brief light stimulation induced action potential–driven Ca^2+^ transients, whereas longer light stimulations prolonged the systolic elevated Ca^2+^ concentration, thereby validating that prolonged depolarization affects Ca^2+^ handling in cardiomyocytes. Since then, a large body of work has used optogenetic pacing *in vitro*, *ex vivo*, and even *in vivo* in anesthetized animals [42, 70–75]. Besides optical pacing, multiple studies have shown that activation of ChR2 in ventricular cardiomyocytes can terminate ventricular tachycardia, even with single-light pulses [76–79]. In parallel, new ChR variants have been established in optogenetics, including engineered channels (mutated and chimeric channels) and newly identified natural ChR. Of particular relevance for perturbing intracellular signaling is the development of channels with increased Ca^2+^ conductance such as CatCh and CapChR2 (ChR2 mutants), which additionally show increased light sensitivity and accelerated response times compared to wild-type ChR2 [80, 81]. Further ground-breaking developments include the discovery and optogenetic application of anion ChR, and, more recently, kalium rhodopsins (see below) [82, 83]. An overview of optogenetic actuators and their respective applications is provided in Table 1 and Fig. 2.

**Table 1:** Optogenetic tools and their application areas in cardiac optogenetic research

A: Optogenetic tools used in cardiac research		
Type	Applied tools	References
Depolarizing optogenetic actuators	ChR2, ChR2(H134R), CatCh, CapChR2, GtACR1	[37, 39, 58, 80–82, 84]
Hyperpolarizing optogenetic actuators	Halo, ArchT, Jaws, PAC-K, WiChR	[67–69, 85–87]
Optogenetic sensors (GECI, GEVI)	GCamP2, GCamP6f, GECO, Cameleon, VSFP2.3, ArcLight, QuasAr, Voltron635	[49–52]
Photoswitchable ligands	Caged-carvedilol, pAzo-1, pAzo-2, Opto-prop-2, PAI, FKF1-GI, optovin, Opto-RGK	[88–95]
Heterologously expressed, unmodified opsins	JellyOp, LWO, OPN4, OPN5	[96–101]
Second messenger optogenetic tools	bPAC, euPAC, OaPAC	[102–105]
B: Application areas in cardiac research		
Application area	Applied tools	References
Optical pacing	ChR2, ChR2(H134R), GtACR1, GtACR2	[39, 42, 44, 70–75, 82, 106–112]
Arrhythmia termination	ChR2, ChR2(H134R), PAC-K, ArchT	[73, 76–79, 84, 86, 109, 113–116]
All-optical electrophysiology	Actuators: ChR2, ChR2(H134R), CatCh, ArchT Sensors: QuasAr1, GCamP6f, VSFP2.3	[55, 57, 59–61, 64–66]
High throughput screening and drug discovery	OptoDyCe, Optopatch, LiEp, OPN5	[55, 57, 64, 65, 71, 101, 117, 118]
Cell-specific control: Neurocardiology	ChR2, ChR2(H134R)	[119–126]
Cell-specific control and imaging: other non-myocytes	ChR2, ChR2(H134R), Arch(T), VSFP2.3	[127–133]
Reviews		[62, 63, 134–142]

Besides applying ChR2 to optically control cardiomyocyte electrophysiology, pioneering work targeted ChR2 to catecholaminergic (sympathetic nervous system) [123] or cholinergic (parasympathetic nervous system) neurons [124], to specifically modulate cardiac signaling through either branch of the autonomic nervous system, commonly referred to as neurocardiology. Sudden increases in heart rate and contractility were observed following light stimulation of sympathetic neurons. This is consistent with the physiological response upon activation of the β1- and β2-AR signaling pathways [121]. Inversely, photostimulation of ChR2-expressing cholinergic neurons in the right atria [120] resulted in prolonged RR intervals, consistent with the activation of cholinergic M2 receptors in the sinus node. Prolonged stimulation (> 30 min) maintained low heart rates, making this a viable approach for control of heart rate *in vivo* [119]. Other work demonstrated that optogenetic stimulation of autonomic neurons of the vagus nerve decreased heart rate [122].

Owing to the heart’s high energy demand for ensuring continuous pump function, cardiomyocytes have the highest density of mitochondria of all cell types examined. A ChR2 variant called mitoChR2 has been expressed at the inner membrane of the mitochondria, providing light-dependent control of the mitochondrial membrane potential, and coupled physiological functions, including Ca^2+^ dynamics [143]. Mitochondria-targeted ChR2 and ChR2-SSFO (a ChR2 mutant with very long opening time) were used to control spontaneous beating of neonatal cardiomyocytes. Prolonged depolarization of the inner mitochondrial membrane with blue light lead to a suppression of spontaneous cardiomyocyte beating, based on the depletion of the mitochondrial membrane gradient (Fig. 2).

Finally, cardiomyocytes make up the largest volume of myocardial tissue, but in terms of numbers, they account for less than half of the cells. Besides exploiting the spatiotemporal benefits of optogenetics by expressing optogenetic tools in cardiomyocytes, various other cell types can be targeted by optogenetics. This includes fibroblasts, macrophages, or endothelial cells. For instance, optogenetics has been used to modulate conduction in the distal AV node by ChR2-based depolarization of electrotonically coupled macrophages [133]. In cell culture systems, ChR2-expressing fibroblasts were used to pace cardiomyocytes at different frequencies following illumination [127, 130, 132], as recently also proposed for whole-hearts post-myocardial infarction [131]. Making use of cell-specific targeting of VSFP2.3, electrical coupling between non-myocytes and cardiomyocytes was demonstrated in the border zone of cryoablation scars [128].

Only recently, robust tools for suppression of cardiac action potentials have been identified. Early on, cardiac research adopted ArchT and NpHR, an outward-directed proton pump and an inward-directed chloride pump, hyperpolarizing the membrane upon photon absorption. A major limitation of these pump-based tools is their small photocurrents, as maximally one ion is transported per photon. Anion ChR, such as GtACR1, can potently inhibit cardiomyocyte action potentials and contraction, but similar to inhibition by ChR2, the inhibitory effect of GtACR1-mediated Cl^−^ currents is achieved by membrane depolarization, rather than hyperpolarization [82]. Improved hyperpolarizing tools include Jaws [85], a red-shifted chloride pump which enables large-volume optogenetic inhibition, and PAC-K, a two-component silencer consisting of photoactivated AC (PACs) and the small cyclic nucleotide-gated potassium channel SthK [86]. While PAC-K efficiently silences cardiomyocyte activity without change in diastolic membrane potential, PAC-K application is limited by slow off-kinetics and use of cAMP, a universal second messenger. Recently, the class of naturally occurring K^+^-selective ChR, so-called kalium rhodopsins, was discovered [83]. Out of these, WiChR, from *Wobblia lunata*, achieves an unprecedented selectivity for K^+^ over Na^+^, and was shown to inhibit spontaneous activity of human-induced pluripotent stem cell–derived atrial cardiomyocytes (Fig. 2) [87].

#### Opsin-GPCR to modulate cardiac G-protein signaling axes

More recently, tools to selectively manipulate and study GPCR pathways have been implemented in cardiac optogenetics with great success, allowing one to target singular components of the signaling pathways. GPCR are heterotrimeric, guanine-nucleotide binding proteins that constitute the largest family of membrane receptors in the human genome. GPCR act via intracellular heterotrimeric G proteins, which can be classified into four families based on their α-subunit: Gs, Gi, Gq, and G12 [144, 145]. Once bound and activated by the GPCR, the α-subunit dissociates and exerts its downstream signaling effect. While the Gs- and Gi-subunits control intracellular cAMP levels via AC by either stimulating or inhibiting AC, respectively, the Gq-subunit activates phospholipase C (PLC) elevating intracellular Ca^2+^ levels. Finally, the G12 subunits stimulate G proteins belonging to the family of Rho kinases.

**Fig. 3:**
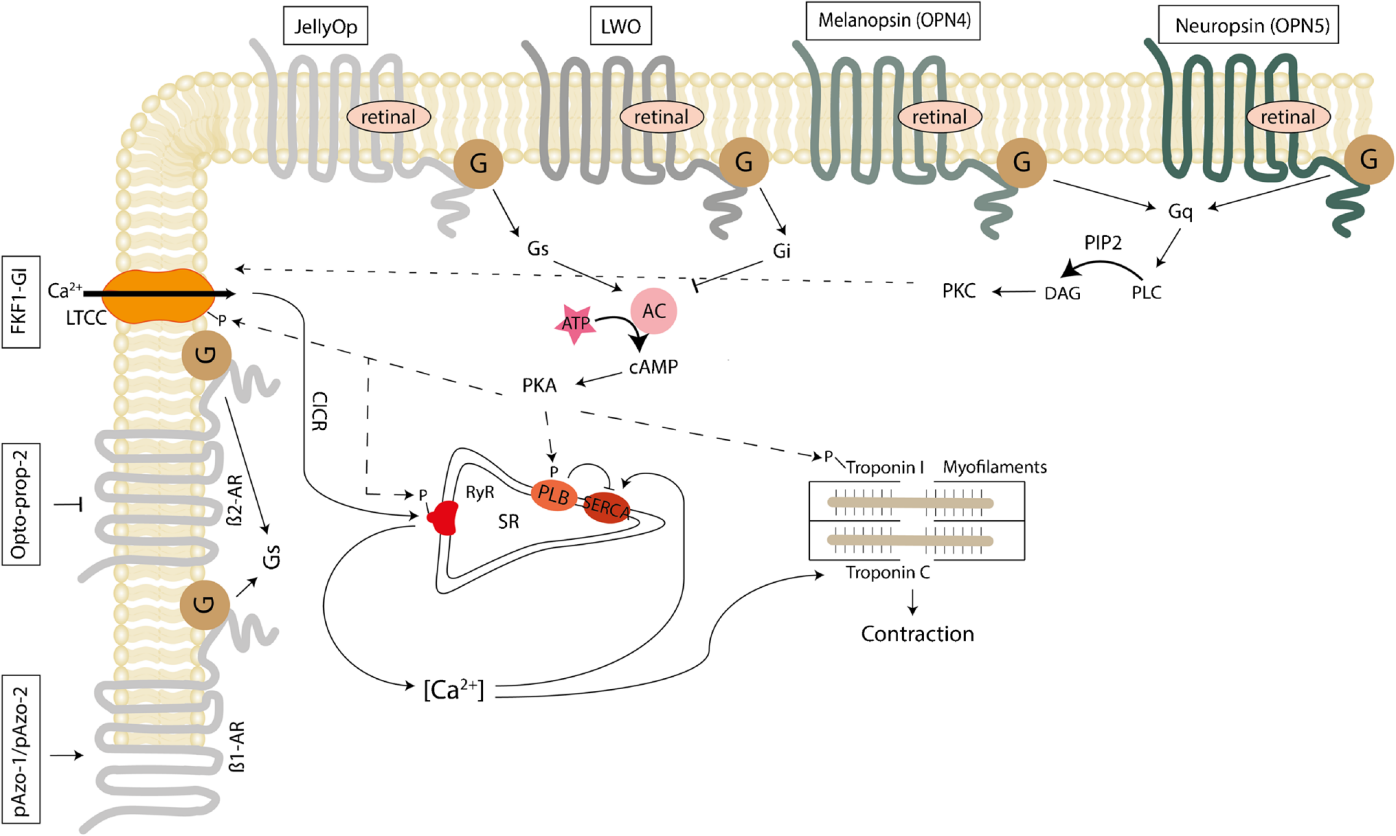
Overview on the GPCR tools used in cardiac research. The GPCR tools that have been applied to cardiac cells and their G-protein binding partners are shown. These include the photoswitchable ligands pAzo-1 and pAzo-2, Opto-prop-2, and FKF1-GI. pAzo-1 and pAzo-2 act as agonists to the β1 adrenergic receptor (β1-AR), thereby activating the Gs-signaling pathway. Opto-prop-2 acts as an antagonist to the β2-AR, inhibiting the Gs-signaling pathway. FKF1-GI acts directly upon L-type Ca^2+^ channels (LTCC). Heterogeneously expressed opsins that have been introduced into cardiomyocytes are also shown: JellyOp (stimulates the Gs-pathway), long-wave opsin (LWO) (activates the Gi-pathway), melanopsin, and neuropsin (acting on the Gq pathway). AC, adenylyl cyclase; ATP, adenosine triphosphate; cAMP, cyclic AMP; CICR, calcium-induced calcium release; DAG, diacylglycerol; PIP2, phosphatidylinositol 4,5-bisphosphate; PKA, protein kinase A; PKC, protein kinase C; PLB, phospholamban; PLC, phospholipase C; RyR, ryanodine receptor; SERCA, SR Ca^2+^-ATPase; SR, sarcoplasmatic reticulum

In the heart, direct optical control of GPCR signalling has been achieved by an optogenetic approach based on photoswitchable-tethered ligands. These were originally developed for activating ionotropic receptors by light and used synthetic photoisomerizable azobenzene as photoswitch. To achieve optical control of channel activity, azobenzenes were coupled to selective ion channel blockers covalently conjugated to the channel proteins [146, 147]. Transfering this approach to GPCR modulation, the irreversible photoswitch caged-carvedilol [89] and the reversible, para-substituted azobenzenes, named parazolol-1 (pAzo-1) and parazolol-2, which specifically target the β1-AR [88] (Fig. 3), were applied to cultured cardiomyocytes, murine-isolated perfused hearts, and living zebrafish larvae. When animals were exposed to pAzo-2 and to 380-nm-light, for example, the measured heart rate was elevated via Gs-signaling, whereas illumination with green light at 550 nm produced a rapid decrease in heart rate. Recently, a novel photoswitchable ligand selectively binding to the β2-AR was derived from the β-blocker propranolol, named, Opto-prop-2. Of note, Opto-prop-2 shows the largest optically induced change in binding affinities (587-fold) recorded so far for synthetic photoswitches modulating GPCR activity [90] (Fig. 3). Chemooptogenetic targeting of the M2 muscarinic receptor was achieved by an engineered molecule called PAI (phthalimide-azo-iperoxo), introducing an azobenzene core into the structure of the M2 muscarinic receptor agonist P-8-Iper. *In vitro* assays revealed light-dependent binding of PAI to M2 receptors, inducing both a decrease in heart rate and prolongation of the AV conduction time [91], in line with native M2 receptor activation by acetylcholine. These experiments highlight the potential of reversible perturbation of adrenergic and cholinergic signaling in the heart, based on optical activation of photoswitchable ligands.

Photoswitches have also been employed to study Ca^2+^ signaling. For instance, Dixon and colleagues used a combination of optogenetics, imaging, and electrophysiology to study the concerted, functional coupling of voltage-gated Ca^2+^ channels [92] (Fig. 3). They applied a light-activated fusion system composed of two components (flavin binding, kelch repeat, F box 1 and Gigantea, FKF1-GI) which bind upon illumination. Using this system, they demonstrated that Ca_v_1.2 channels form clusters in ventricular myocytes and that the interaction between these channels via their C-tails leads to coordinated gating, thereby increasing Ca^2+^ influx and ECC. This is especially interesting for assessing mechanisms underlying arrhythmogenesis in patients with Timothy syndrome, where mutated Ca_v_1.2 channels can increase the activity of coupled wildtype channels, thereby increasing Ca^2+^ currents, diastolic and systolic Ca^2+^ levels, contractility, and the frequency of arrhythmogenic Ca^2+^ fluctuations in ventricular myocytes. In a different approach, the light-activatable ligand optovin has been shown to activate endogenous Trpa1b channels upon violet illumination in zebrafish [93]. In embryonic zebrafish overexpressing Trpa1a in cardiomyocytes and treated with optovin, this has been used for optical pacing *in vivo* [94]. Another group showed that an engineered Ras-like GTPase, a negative regulator of voltage-gated Ca^2+^ channels, could effectively attenuate rhythmic Ca^2+^ oscillations upon blue light application in cardiomyocyte-like HL-1 cells [95].

The potential for creating photoswitchable drug-like compounds to target specific signaling pathways is enormous, since it can be applied to essentially every agonist/antagonist-receptor pair. Additionally, most strategies for cardiac rhythm control depend on antiarrhythmic drugs that primarily target specific ionic currents. However, the effects of these drugs cannot be precisely regulated in respect to target organ, cell type, and time window of action, frequently leading to undesirable side effects, such as life-threatening ventricular pro-arrhythmogenicity. Photopharmacology offers the unique benefit of remote spatiotemporal control of physiological processes, while simultaneously utilizing light-sensitive, exogenous small molecules that can be tested and approved using standard drug development procedures (in contrast with other optogenetic approaches, which rely on heterologous expression of photoreceptors and delivery vectors that risk an immune response).

Another approach towards optical dissection of GPCR signaling is the expression of naturally occurring, unmodified opsins in target cells. Contrary to photoswitches, ectopically expressed opsins typically require no addition of exogenous ligands, but bind available retinal isomers. In comparison to traditional microbial optogenetic tools, which are ion channels and ion pumps, rhodopsin GPCR benefit from the intrinsic signal amplification cascade by cycling the G protein, increasing their light sensitivity by more than three log units [148].

Various rhodopsins have been expressed in cardiomyocytes. For instance, the Gs protein coupled box jellyfish opsin (JellyOp) was introduced into cardiomyocytes and triggered optogenetic stimulation of Gs-signaling in both, isolated cardiomyocytes, and the whole heart (Fig. 3). Illumination stimulated an increase in cAMP levels and accelerated spontaneous beating rates consistent with the response following pharmacological β-adrenergic stimulation. Additionally, illumination of isolated ventricular cardiomyocytes evoked an instantaneous increase of L-type Ca^2+^ currents, a known consequence of β-AR signaling. Finally, activation of JellyOp also had a pronounced effect on relaxation speed, similar to the increase in lusitropy after β-adrenergic stimulation [96].

On the contrary, long-wave cone opsin, stimulating Gi and lowering intracellular cAMP levels, was shown to diminish L-type Ca^2+^ currents by reducing PKA activity [97] (Fig. 3). In certain cardiac cells, such as atrial myocytes as well as nodal pacemaker cells, acetylcholine triggers the activation of M2-muscarinic acetylcholine receptors and, consequently, the opening of GIRK channels, via its Gi-βγ subunits. This results in membrane hyperpolarization, a decrease in heart rate, prolongation of AV-node conduction, and action potential shortening in atrial cardiomyocytes. Stimulation of long wave opsin in these cells produced the same effects [97].

Besides rhodopsins that modulate the cAMP signaling pathway, melanopsin (OPN4) [98] and neuropsin (OPN5) [100, 101], both activating the Gq pathway, have been expressed in cardiac cells, where optical activation of Gq signaling induced positive inotropic and chronotropic effects (Fig. 3). Specifically, UV-light triggered OPN5 and Gq activation induced IP3 generation and Ca^2+^ transients and inhibited GIRK channel activity. In a different study, melanopsin was applied to optogenetically generate Ca^2+^ oscillations upon pulsed illumination, which was utilized to understand the relative contributions of calcium oscillation frequency, amplitude, and duty cycle to transcriptional activity of the calcium-dependent transcription factor NFAT [99]. This is especially interesting, since NFAT activity is a critical component of the antiapoptotic pathway that regulates whether the outcome of calcineurin activation is cardiomyocyte apoptosis or survival. Dysregulation of this pathway often results in cardiomyocyte dysfunction, apoptosis, and ultimately heart failure [149]. Additionally, OPN5 has been applied as an all-optical high-throughput screening technology for TRPC6 inhibitors and was shown to be more sensitive and specific than established pharmacological screenings [101]. TRPC6 channels are important drug targets that are physiologically activated by DAG following Gq activation and the stimulation of PLCβ, which play a role in many diseases, including pulmonary hypertension. Traditionally, the screening of drugs that could potentially inhibit TRPC6 channels involved physiologically stimulating Gq/PLCβ signaling with acetylcholine or ATP. In their high throughput-screening assay, Wagdi and colleagues replaced acetylcholine addition by light activation of hOPN5, to activate the downstream Gq/PLCβ signaling pathways. However, when rhodopsins are exogenously expressed, they do not necessarily optimally interact with the native G proteins in the target cell: there are many subtypes within each G-protein family; many GPCR are promiscuous with a particular G protein activation “fingerprint,” and not each cell type expresses the same complement of regulator of G-protein signaling (RGS) proteins, including G-protein receptor kinases (GRK) and arrestins.

#### Second messenger optogenetic tools

Bypassing the receptor activation and directly targeting down-stream GPCR effectors or regulators allows the probing of individual signaling components. This is useful as distinct GPCRs can activate converging downstream-signaling, or, conversely, trigger distinct physiological responses despite generating similar levels of second messengers, such as cAMP. For example, the β1-AR and ET-1 both elicit a similar increase in cAMP, but only β1-AR activation results in positive inotropy in cardiomyocytes. This is the case even for receptors within the same subfamily: even though both β1-AR and β2-AR couple to Gs proteins and trigger cytosolic cAMP elevation, they were shown to have distinct effects on cardiac function. To address this conundrum, Lin and colleagues employed a bacterial photoactivated AC, bPAC from *Beggiatoa*, to demonstrate that cardiomyocytes distinguish between cAMP levels in different subcellular compartments, with cAMP increase in individual compartments eliciting distinct physiological outputs [150]. Their findings demonstrate that the production of cAMP from the Golgi results in the regulation of a PKA target that accelerates the rate of cardiomyocyte relaxation, while cAMP generation from the plasma membrane activates a distinct PKA that promotes an increase in contractile force. Other examples of natural PAC variants include euPAC from *Euglena gracilis* [103] and OaPAC from *Oscillatoria acuminate* [105]. Recently, a variety of red-shifted light-activatable AC have also been engineered that can be regulated by near-infrared light; however, these have yet to be applied in the cardiac system [151–153].

Besides cAMP, cGMP levels can be optogenetically manipulated using microbial enzyme rhodopsins. Enzyme rhodopsins are a class of natural rhodopsin-based photoreceptors that possess light-regulated enzyme activity. These enzyme rhodopsins include histidine kinase rhodopsins, rhodopsin phosphodiesterases, and rhodopsin guanylyl cyclases (Rh-GC) [154]. BeGC1 is a Rh-GC derived from the aquatic fungus *Blastocladiella emersonii*. Hagio and colleagues applied both BeGC1 and several PAC variants to study effects of cyclic nucleotide levels in cardiomyocytes. While blue-light activation of bPAC for 5 s gradually reduced heart rate, light stimulation of cardiomyocytes expressing BeGC1 or OaPAC induced neither cardiac arrest nor bradycardia, suggesting that bPAC remains the superior tool for cardiac application [155].

Finally, cAMP and cGMP levels can be optogenetically modulated by increasing their respective hydrolysis through light-activatable phosphodiesterases [156], yet to be tested for studying compartment-specific cyclic nucleotide signaling in cardiomyocytes.

The other main second messenger within the heart, Ca^2+^, has also been the target of optogenetic manipulation itself. Optogenetic tools have been developed to modulate the intracellular Ca^2+^ level and include PACR [157], Opto-CRAC [158], and Opto-STIM1 [159]. However, these approaches have not been applied to the cardiovascular system yet.

## Perspectives

As we described, a wide variety of optogenetic tools have been applied to study diverse aspects of cardiac signaling, from top-down regulation through the sympathetic and parasympathetic nervous system, tapping into the signaling pathways, to targeting subcellular compartment-specific processes in cardiomoycytes. The main research focus has been on creating photoactivatable agonists and antagonists of cardiac receptor pathways, which provide advantages over conventional pharmacological approaches, such as β-blockers. Benefits include higher receptor subtype specificity, cell-type specificity, and increased spatiotemporal control over delivery and activation.

- 1. Exploiting the GPCR-signaling system using optogenetics

Cardiac activity is heavily regulated by GPCR signaling, bringing promise to natural rhodopsins acting as GPCR (type II rhodopsins) and chimeric Opto-GPCR as optimal interrogators of cardiac function, as research tools and as potential therapeutics to replace pharmacological agonists and antagonists of GPCR activation. Chimeric Opto-GPCR combine the light sensitive moiety of an opsin GPCR with the G protein–interacting sites, e.g., the intracellular domains, of a target receptor. Opto-GPCR were developed as light-sensitive surrogates for the target receptor and its particular signaling cascade. By introducing the G-protein–binding domains of the target receptor, the chimeric protein would then couple to the native G protein of the target receptor, essentially hijacking the specific target signaling pathway. The first Opto-GPCR, chimeric proteins between bovine rhodopsin and β2-AR, were created in 2005 by the Khorana team [160]. Since then, the original approach of replacing all intracellular loops and the C-terminus has been optimized, and it is now known that only the intracellular loop 3 and the C-terminus are sufficient to activate the desired downstream signaling pathways [161, 162]. To date, Opto-GPCR have been exclusively tested in neural systems and have yet to be applied to the cardiovascular system. However, it is easy to conceive the enormous potential that these Opto-GPCR possess. Both the adrenergic and the M2 muscarinic receptors present attractive targets for chimeric Opto-GPCR, but so far, only Opto-AR have been generated. In the future, applying matching chimeric receptors to the cardiovascular signaling pathways can grant further insight into precise signaling mechanisms of individual receptor subtypes in physiological and pathophysiological conditions, and, using multi-color optogenetic approaches, illuminate cross-talk between GPCR-triggered signaling pathways. This is especially interesting for receptor subtypes for which cardiac function is not fully understood. For instance, it is still unclear to what degree and in which manner the β3-AR is implicated in heart failure. β3-AR, only making up 3% of cardiac β-AR, were shown to act as a “fuse” against cardiac adrenergic overstimulation by producing negative inotropy and are differentially expressed in the healthy and diseased heart. Different preclinical studies indicate that β3-AR activation may have cardio-protective effects similar to β-blockers [163, 164]. Employing an Opto-GPCR mimicking β3-AR signaling could help researchers to determine the underlying signaling events.

The GPCR regulating systems also include GRK and β-arrestins that terminate G-protein signaling, and both were targeted with optogenetic tools. For instance, an optogenetic β-arrestin-2 was built based on the cryptochrome CRY2-CIB system. In detail, the CRY2 component was fused to the N-terminus of β-arrestin, while CIB was fused to the C-terminus of the β2-AR [165]. CRY2 is a blue-light receptor from *A. thaliana* that uses flavin adenine dinucleotide as chromophore, which, upon blue light exposure, dimerizes and binds to its native partner, CIB [166]. Light-induced CRY2-CIB interaction induced binding of β-arrestin-2 to the β2-AR, followed by efficient receptor endocytosis. Applying this system to cardiomyocytes would be of particular interest in pathophysiological states, to further investigate the role of β-arrestins in the attenuation of β-AR signaling following the excessive sympathetic stimulation during heart failure.

Other GPCR regulatory proteins are involved in the desensitization of GPCRs observed during heart failure, providing additional targets for potential interventions. One study utilized a similar system as above to design and employ an optogenetic RGS, called opto-RGS, rendering the RGS photoactivatable and terminating Gαq-induced Ca^2+^ signaling mediated by the activation of the acetylcholine receptor, M3R [167]. RGS proteins are GPCR regulators that accelerate the GTPase activity of the Gα subunit, thereby facilitating the re-association with the Gβγ subunits. The CRY2-CIB system has also been applied to a GRK, which couples to and sequesters the Gβγ subunits [168].

Finally, an alternative to the optical activation of such heterologous constructs is optogenetic kinase inhibitors that enable the silencing of native kinases. These light-regulated kinase inhibitors are based on the light-oxygen-voltage-sensing (LOV2) photosensory domain and include tools such as Opto-JNKi to control the MAPK pathway [169] or Opto-PKI, which inhibits PKA [170]. While optogenetic control of kinases has not been applied explicitly in the context of cardiac signaling, this toolkit enables the probing of the roles of specific pathways in a component-specific manner.

- 2. Optogenetic approaches to study Ca^2+^ signaling

Most of the optogenetic actuators targeting Ca^2+^ signaling were developed to perturb intracellular Ca^2+^ levels in non-myocytes and cellular processes outside of the heart [171]. Whereas one direction of interest was the development of calcium-permeable ChR mutants for optical control of Ca^2+^ signaling (CapChR2 [81], CatCh [80]), others focused on engineering Ca^2+^ channels that can be directly targeted by light (Opto-STIM1 [159, 172], monSTIM1 [173], Opto-CRAC [158], BACCS [174], eOS1 [175], LOCa [176]). While none of the underlying channels are involved in native ECC in the heart, they highlight the potential of directly targeting the involved Ca^2+^ channels, which could conceptually be transferred to LTCC.

The modern optogenetic toolkit permits insight into subcellular mechanisms involved in ECC in a hitherto inconceivable manner. This benefit should be exploited further, thereby granting a better understanding of how the individual subcellular components of the ECC machinery may be dysregulated in cardiac disease. For instance, it would be very useful if proteins involved in CICR could be targeted by light, such as the RyR, SERCA, or PLB. Importantly, the downstream messenger proteins and kinases involved in Ca^2+^ dynamics, calmodulin or CaMKII, were directly targeted using optogenetic manipulation. Firstly, an optogenetic inhibitor for CaMKII, called paAIP2, inhibits CaMKII activity upon illumination. PaAIP2 is based on the fusion of a blue-light–sensitive LOV domain to the autocamtide inhibitory peptide 2 (AIP2) [177]. In a different study, calmodulin was engineered to directly manipulate subcellular Ca^2+^ concentration upon light stimulation. The photoactivatable Ca^2+^-releasing protein, PACR, was developed by inserting the LOV2 domain into the calcium-binding protein, calmodulin, fused to the M13 peptide [157]. Applying these tools to cardiac cells will provide insight into the downstream mechanisms of Ca^2+^ handling and in turn aid in optimizing therapeutic approaches.

- 3. Applications of optogenetic signaling tools

Optogenetic studies that tackle open problems in cardiovascular pathophysiology are still rare. Proposed therapeutic approaches for heart rhythm control, such as optical pacing and arrhythmia termination, utilize light-driven ion pumps and, more frequently, light-gated ion channels. The underlying microbial rhodopsins are, however, potentially immunogenic and require very high light intensities (1·10^16^ photons cm^-2^ s^-1^). Reaching such light intensities within human hearts would require multi-site intracardiac light delivery, at least when using blue light for rhodopsin activation, and might be phototoxic [178]. Furthermore, cardiac cells are not simple “on–off” systems, but instead, their electromechanical activity is strongly regulated depending on required cardiac output, including important regulation by GPCR. In combination with viral delivery and cell-type–specific promoters, the Opto-GPCR technology therefore holds great promise for investigating cardiac regulation in health and disease, paving the path to the development of cell-tailored therapeutics. Nonetheless, in order to establish optogenetic therapy to be used in the clinics, safe and effective gene therapy and light delivery remain two major hurdles that need to be overcome.

Finally, multiple high throughput electrophysiology platforms that build upon optogenetic technologies have been introduced, such as OptoDyCE [65], Optopatch [71], or LiEp [57]. Some of these platforms are already commercially available, thereby supporting the entire drug development pipeline at low cost and with high spatiotemporal benefits.

Taken together, the optogenetic toolkit has expanded greatly over the past decade, and scientists have started to apply these novel tools for cardiovascular research. The possibilities of creating and using optogenetic tools are near limitless, and the surface has only been scratched.

## Data Availability

No datasets were generated or analysed during the current study.

## Abbreviations

AC: Adenylyl cyclase
AIP: Autocamide inhibitory peptide
Ang II: Angiotensin II
AR: Adrenergic receptor
Arch(T): Archaerhodopsin
AT-1/AT-2: Angiotensin II receptor 1/2
AV: Atrioventricular
CaMKII: Calmodulin-dependent protein kinase II
cAMP: Cyclic adenosine monophosphate
cGMP: Cyclic guanosine monophosphate
ChR: Channelrhodopsin
CICR: Calcium-induced calcium release
ECC: Excitation-contraction coupling
ET-1: Endothelin-1
ET-A/ET-B: Endothelin-1 receptor A/B
GC: Guanylyl cyclase
GECI: Genetically encoded calcium indicator
GEVI: Genetically encoded voltage indicator
GPCR: G-protein coupled receptor
GRK: G-protein receptor kinase
hERG: Voltage-gated K^+^ channel subfamily H member 2
IP3: Inositol-1,4,5-trisphosphate
LOV: Light-oxygen-voltage-sensing domain
LTCC: L-type Ca^2+^ channel
MAPK: Mitogen-activated protein kinase
NCX: Na^+^/Ca^2+^ exchanger
NpHR: Halorhodopsin
PAC: Photoactivated adenylyl cyclase
PAI: Phthalimide-Azo-Iperoxo
PKA: Protein kinase A
PLB: Phospholamban
PLC: Phospholipase C
RGS: Regulator of G-protein signaling
RyR: Ryanodine receptor
SERCA: Sarcoplasmic/endoplasmic Ca^2+^ ATPase
SR: Sarcoplasmatic reticulum

## References

[1] Litvinukova M (2020). Cells of the adult human heart. Nature.

[2] Bers DM (2002). Cardiac excitation-contraction coupling. Nature.

[3] Eisner DA (2017). Calcium and excitation-contraction coupling in the heart. Circ Res.

[4] Bers DM (2008). Calcium cycling and signaling in cardiac myocytes. Annu Rev Physiol.

[5] Bers DM, Guo T (2005). Calcium signaling in cardiac ventricular myocytes. Ann N Y Acad Sci.

[6] Maier LS, Bers DM (2007). Role of Ca2+/calmodulin-dependent protein kinase (CaMK) in excitation-contraction coupling in the heart. Cardiovasc Res.

[7] Dewenter M (2017). Calcium signaling and transcriptional regulation in cardiomyocytes. Circ Res.

[8] Capote LA, Mendez Perez R, Lymperopoulos A (2015). GPCR signaling and cardiac function. Eur J Pharmacol.

[9] Neuhof C, Neuhof H (2014). Calpain system and its involvement in myocardial ischemia and reperfusion injury. World J Cardiol.

[10] Kumari N, Gaur H, Bhargava A (2018). Cardiac voltage gated calcium channels and their regulation by beta-adrenergic signaling. Life Sci.

[11] Duraes Campos I (2018). A brain within the heart: a review on the intracardiac nervous system. J Mol Cell Cardiol.

[12] Gordan R, Gwathmey JK, Xie LH (2015). Autonomic and endocrine control of cardiovascular function. World J Cardiol.

[13] Bristow MR (1986). Beta 1- and beta 2-adrenergic-receptor subpopulations in nonfailing and failing human ventricular myocardium: coupling of both receptor subtypes to muscle contraction and selective beta 1-receptor down-regulation in heart failure. Circ Res.

[14] Myagmar BE (2017). Adrenergic receptors in individual ventricular myocytes: the beta-1 and alpha-1B are in all cells, the alpha-1A is in a subpopulation, and the beta-2 and beta-3 are mostly absent. Circ Res.

[15] Michel LYM, Farah C, Balligand JL (2020) The beta3 adrenergic receptor in healthy and pathological cardiovascular tissues. Cells 9(12):2584 10.3390/cells9122584 PMC7761574 33276630

[16] de Lucia C, Eguchi A, Koch WJ (2018). New insights in cardiac beta-adrenergic signaling during heart failure and aging. Front Pharmacol.

[17] Lymperopoulos A, Rengo G, Koch WJ (2013). Adrenergic nervous system in heart failure: pathophysiology and therapy. Circ Res.

[18] Lissandron V, Zaccolo M (2006). Compartmentalized cAMP/PKA signalling regulates cardiac excitation-contraction coupling. J Muscle Res Cell Motil.

[19] Mangoni ME, Nargeot J (2008). Genesis and regulation of the heart automaticity. Physiol Rev.

[20] Yaniv Y (2015). Real-time relationship between PKA biochemical signal network dynamics and increased action potential firing rate in heart pacemaker cells: kinetics of PKA activation in heart pacemaker cells. J Mol Cell Cardiol.

[21] Fedorov VV (2011). Anatomic localization and autonomic modulation of atrioventricular junctional rhythm in failing human hearts. Circ Arrhythm Electrophysiol.

[22] Mazgalev TN (1999). Autonomic modification of the atrioventricular node during atrial fibrillation: role in the slowing of ventricular rate. Circulation.

[23] Conti V (2013). Adrenoreceptors and nitric oxide in the cardiovascular system. Front Physiol.

[24] Calvert JW (2011). Exercise protects against myocardial ischemia-reperfusion injury via stimulation of beta(3)-adrenergic receptors and increased nitric oxide signaling: role of nitrite and nitrosothiols. Circ Res.

[25] Schlossmann J, Feil R, Hofmann F (2003). Signaling through NO and cGMP-dependent protein kinases. Ann Med.

[26] Harvey RD (2012). Muscarinic receptor agonists and antagonists: effects on cardiovascular function. Handb Exp Pharmacol.

[27] Silvani A et al (2016) Brain-heart interactions: physiology and clinical implications. Philos Trans A Math Phys Eng Sci 374(2067):20150181 10.1098/rsta.2015.0181 27044998

[28] Frey N, Olson EN (2003). Cardiac hypertrophy: the good, the bad, and the ugly. Annu Rev Physiol.

[29] Shah R (2007). Endothelins in health and disease. Eur J Intern Med.

[30] Drawnel FM, Archer CR, Roderick HL (2013). The role of the paracrine/autocrine mediator endothelin-1 in regulation of cardiac contractility and growth. Br J Pharmacol.

[31] Houde M, Desbiens L, D'Orleans-Juste P (2016). Endothelin-1: biosynthesis, signaling and vasoreactivity. Adv Pharmacol.

[32] Sadoshima J (1993). Autocrine release of angiotensin II mediates stretch-induced hypertrophy of cardiac myocytes in vitro. Cell.

[33] Yamazaki T (1996). Endothelin-1 is involved in mechanical stress-induced cardiomyocyte hypertrophy. J Biol Chem.

[34] Khorana HG (1979). Amino acid sequence of bacteriorhodopsin. Proc Natl Acad Sci U S A.

[35] Khorana HG (1988). Expression of a bovine rhodopsin gene in Xenopus oocytes: demonstration of light-dependent ionic currents. Proc Natl Acad Sci U S A.

[36] Nagel G (2002). Channelrhodopsin-1: a light-gated proton channel in green algae. Science.

[37] Nagel G (2003). Channelrhodopsin-2, a directly light-gated cation-selective membrane channel. Proc Natl Acad Sci U S A.

[38] Boyden ES (2005). Millisecond-timescale, genetically targeted optical control of neural activity. Nat Neurosci.

[39] Bruegmann T (2010). Optogenetic control of heart muscle in vitro and in vivo. Nat Methods.

[40] Deisseroth K (2006). Next-generation optical technologies for illuminating genetically targeted brain circuits. J Neurosci.

[41] Deisseroth K (2015). Optogenetics: 10 years of microbial opsins in neuroscience. Nat Neurosci.

[42] Arrenberg AB (2010). Optogenetic control of cardiac function. Science.

[43] Hofmann B (2010). Light induced stimulation and delay of cardiac activity. Lab Chip.

[44] Jia Z (2011). Stimulating cardiac muscle by light: cardiac optogenetics by cell delivery. Circ Arrhythm Electrophysiol.

[45] Morad M, Salama G (1979). Optical probes of membrane potential in heart muscle. J Physiol.

[46] Golovina VA, Blaustein MP (1997). Spatially and functionally distinct Ca2+ stores in sarcoplasmic and endoplasmic reticulum. Science.

[47] Kendall JM (1996). Recombinant apoaequorin acting as a pseudo-luciferase reports micromolar changes in the endoplasmic reticulum free Ca2+ of intact cells. Biochem J.

[48] Ottolini D, Cali T, Brini M (2014). Methods to measure intracellular Ca(2+) fluxes with organelle-targeted aequorin-based probes. Methods Enzymol.

[49] Miyawaki A (1997). Fluorescent indicators for Ca2+ based on green fluorescent proteins and calmodulin. Nature.

[50] Tallini YN (2006). Imaging cellular signals in the heart in vivo: Cardiac expression of the high-signal Ca2+ indicator GCaMP2. Proc Natl Acad Sci U S A.

[51] Nakao S, Wakabayashi S, Nakamura TY (2015). Stimulus-dependent regulation of nuclear Ca2+ signaling in cardiomyocytes: a role of neuronal calcium sensor-1. PLoS One.

[52] Shang W (2014). Imaging Ca2+ nanosparks in heart with a new targeted biosensor. Circ Res.

[53] Sanchez C et al (2021) Detection of Ca2+ transients near ryanodine receptors by targeting fluorescent Ca2+ sensors to the triad. J Gen Physiol 153(4):e202012592 10.1085/jgp.202012592 PMC7868779 33538764

[54] Pahlavan S, Morad M (2017). Total internal reflectance fluorescence imaging of genetically engineered ryanodine receptor-targeted Ca(2+) probes in rat ventricular myocytes. Cell Calcium.

[55] Hochbaum DR (2014). All-optical electrophysiology in mammalian neurons using engineered microbial rhodopsins. Nat Methods.

[56] Abdelfattah AS (2019). Bright and photostable chemigenetic indicators for extended in vivo voltage imaging. Science.

[57] Streit J, Kleinlogel S (2018). Dynamic all-optical drug screening on cardiac voltage-gated ion channels. Sci Rep.

[58] Nagel G (2005). Light activation of channelrhodopsin-2 in excitable cells of Caenorhabditis elegans triggers rapid behavioral responses. Curr Biol.

[59] Miesenbock G, Kevrekidis IG (2005). Optical imaging and control of genetically designated neurons in functioning circuits. Annu Rev Neurosci.

[60] Zemelman BV (2002). Selective photostimulation of genetically chARGed neurons. Neuron.

[61] Zemelman BV (2003). Photochemical gating of heterologous ion channels: remote control over genetically designated populations of neurons. Proc Natl Acad Sci U S A.

[62] Miesenbock G (2009). The optogenetic catechism. Science.

[63] Grosenick L, Marshel JH, Deisseroth K (2015). Closed-loop and activity-guided optogenetic control. Neuron.

[64] Hou JH (2014). Simultaneous mapping of membrane voltage and calcium in zebrafish heart in vivo reveals chamber-specific developmental transitions in ionic currents. Front Physiol.

[65] Klimas A (2016). OptoDyCE as an automated system for high-throughput all-optical dynamic cardiac electrophysiology. Nat Commun.

[66] Scardigli M (2018). Real-time optical manipulation of cardiac conduction in intact hearts. J Physiol.

[67] Zhang F (2007). Multimodal fast optical interrogation of neural circuitry. Nature.

[68] Gradinaru V, Thompson KR, Deisseroth K (2008). eNpHR: a Natronomonas halorhodopsin enhanced for optogenetic applications. Brain Cell Biol.

[69] Han X (2011). A high-light sensitivity optical neural silencer: development and application to optogenetic control of non-human primate cortex. Front Syst Neurosci.

[70] Ambrosi CM, Entcheva E (2014). Optogenetics' promise: pacing and cardioversion by light?. Future Cardiol.

[71] Dempsey GT (2016). Cardiotoxicity screening with simultaneous optogenetic pacing, voltage imaging and calcium imaging. J Pharmacol Toxicol Methods.

[72] Alex A (2015). Optogenetic pacing in Drosophila melanogaster. Sci Adv.

[73] Nussinovitch U, Gepstein L (2015). Optogenetics for in vivo cardiac pacing and resynchronization therapies. Nat Biotechnol.

[74] Dong R (2019). A protocol for dual calcium-voltage optical mapping in murine sinoatrial preparation with optogenetic pacing. Front Physiol.

[75] Dwenger M (2019). Chronic optical pacing conditioning of h-iPSC engineered cardiac tissues. J Tissue Eng.

[76] Bruegmann T (2016). Optogenetic defibrillation terminates ventricular arrhythmia in mouse hearts and human simulations. J Clin Invest.

[77] Nyns ECA (2017). Optogenetic termination of ventricular arrhythmias in the whole heart: towards biological cardiac rhythm management. Eur Heart J.

[78] Sasse P (2019). Optogenetic termination of cardiac arrhythmia: mechanistic enlightenment and therapeutic application?. Front Physiol.

[79] Quinonez Uribe RA (2018). Energy-reduced arrhythmia termination using global photostimulation in optogenetic murine hearts. Front Physiol.

[80] Kleinlogel S (2011). Ultra light-sensitive and fast neuronal activation with the Ca(2)+-permeable channelrhodopsin CatCh. Nat Neurosci.

[81] Fernandez Lahore RG (2022). Calcium-permeable channelrhodopsins for the photocontrol of calcium signalling. Nat Commun.

[82] Kopton RA (2018). Cardiac electrophysiological effects of light-activated chloride channels. Front Physiol.

[83] Govorunova EG (2022). Kalium channelrhodopsins are natural light-gated potassium channels that mediate optogenetic inhibition. Nat Neurosci.

[84] Bingen BO (2014). Light-induced termination of spiral wave arrhythmias by optogenetic engineering of atrial cardiomyocytes. Cardiovasc Res.

[85] Chuong AS (2014). Noninvasive optical inhibition with a red-shifted microbial rhodopsin. Nat Neurosci.

[86] Bernal Sierra YA (2018). Potassium channel-based optogenetic silencing. Nat Commun.

[87] Vierock J (2022). WiChR, a highly potassium-selective channelrhodopsin for low-light one- and two-photon inhibition of excitable cells. Sci Adv.

[88] Duran-Corbera A (2022). A photoswitchable ligand targeting the beta(1) -adrenoceptor enables light-control of the cardiac rhythm. Angew Chem Int Ed Engl.

[89] Duran-Corbera A (2022). Caged-carvedilol as a new tool for visible-light photopharmacology of beta-adrenoceptors in native tissues. iScience.

[90] Bosma R (2022). Optical control of the beta(2)-adrenergic receptor with opto-prop-2: a cis-active azobenzene analog of propranolol. iScience.

[91] Riefolo F (2019). Optical control of cardiac function with a photoswitchable muscarinic agonist. J Am Chem Soc.

[92] Dixon RE (2012). Ca2+ signaling amplification by oligomerization of L-type Cav1.2 channels. Proc Natl Acad Sci U S A.

[93] Kokel D (2013). Photochemical activation of TRPA1 channels in neurons and animals. Nat Chem Biol.

[94] Lam PY (2017). A high-conductance chemo-optogenetic system based on the vertebrate channel Trpa1b. Sci Rep.

[95] Ma G (2018). Optogenetic control of voltage-gated calcium channels. Angew Chem Int Ed Engl.

[96] Makowka P (2019). Optogenetic stimulation of Gs-signaling in the heart with high spatio-temporal precision. Nat Commun.

[97] Cokić M et al (2021) Optogenetic stimulation of gi signaling enables instantaneous modulation of cardiomyocyte pacemaking. Front Physiol 12(2255):768495 10.3389/fphys.2021.768495 PMC8721037 34987414

[98] Beiert T, Bruegmann T, Sasse P (2014). Optogenetic activation of Gq signalling modulates pacemaker activity of cardiomyocytes. Cardiovasc Res.

[99] Hannanta-Anan P, Chow BY (2016). Optogenetic control of calcium oscillation waveform defines NFAT as an integrator of calcium load. Cell Syst.

[100] Dai R (2022). A neuropsin-based optogenetic tool for precise control of Gq signaling. Sci China Life Sci.

[101] Wagdi A (2022). Selective optogenetic control of G(q) signaling using human neuropsin. Nat Commun.

[102] Stuven B (2019). Characterization and engineering of photoactivated adenylyl cyclases. Biol Chem.

[103] Iseki M (2002). A blue-light-activated adenylyl cyclase mediates photoavoidance in Euglena gracilis. Nature.

[104] Lin T-Y et al (2023) Cardiac contraction and relaxation are regulated by beta 1 adrenergic receptor-generated cAMP pools at distinct membrane locations. bioRxiv, 2022.07.13.499965

[105] Ohki M (2016). Structural insight into photoactivation of an adenylate cyclase from a photosynthetic cyanobacterium. Proc Natl Acad Sci U S A.

[106] Williams JC (2013). Computational optogenetics: empirically-derived voltage- and light-sensitive channelrhodopsin-2 model. PLoS Comput Biol.

[107] Ambrosi CM (2015). Optogenetics-enabled assessment of viral gene and cell therapy for restoration of cardiac excitability. Sci Rep.

[108] Ambrosi CM, Entcheva E (2014). Optogenetic control of cardiomyocytes via viral delivery. Methods Mol Biol.

[109] Nussinovitch U, Gepstein L (2015). Optogenetics for suppression of cardiac electrical activity in human and rat cardiomyocyte cultures. Neurophotonics.

[110] Zgierski-Johnston CM (2020). Cardiac pacing using transmural multi-LED probes in channelrhodopsin-expressing mouse hearts. Prog Biophys Mol Biol.

[111] Kopton RA et al (2020) Electromechanical assessment of optogenetically modulated cardiomyocyte activity. J Vis Exp (157). 10.3791/60490 10.3791/60490 32202521

[112] Schwarzova B et al (2023) Modulating cardiac physiology in engineered heart tissue with the bidirectional optogenetic tool BiPOLES. Pflugers Arch. 10.1007/s00424-023-02869-x 10.1007/s00424-023-02869-x PMC10730631 37863976

[113] Crocini C (2016). Optogenetics design of mechanistically-based stimulation patterns for cardiac defibrillation. Sci Rep.

[114] Govorunova EG (2016). Anion channelrhodopsins for inhibitory cardiac optogenetics. Sci Rep.

[115] Boyle PM (2018). Termination of re-entrant atrial tachycardia via optogenetic stimulation with optimized spatial targeting: insights from computational models. J Physiol.

[116] Funken M (2019). Optogenetic hyperpolarization of cardiomyocytes terminates ventricular arrhythmia. Front Physiol.

[117] Bjork S (2017). Evaluation of optogenetic electrophysiology tools in human stem cell-derived cardiomyocytes. Front Physiol.

[118] Klimas A (2020). Multimodal on-axis platform for all-optical electrophysiology with near-infrared probes in human stem-cell-derived cardiomyocytes. Prog Biophys Mol Biol.

[119] Mighiu AS, Heximer SP (2012). Controlling parasympathetic regulation of heart rate: a gatekeeper role for RGS proteins in the sinoatrial node. Front Physiol.

[120] Moreno A (2019). Sudden heart rate reduction upon optogenetic release of acetylcholine from cardiac parasympathetic neurons in perfused hearts. Front Physiol.

[121] Prando V (2018). Dynamics of neuroeffector coupling at cardiac sympathetic synapses. J Physiol.

[122] Rajendran PS (2019). Identification of peripheral neural circuits that regulate heart rate using optogenetic and viral vector strategies. Nat Commun.

[123] Wengrowski AM (2015). Optogenetic release of norepinephrine from cardiac sympathetic neurons alters mechanical and electrical function. Cardiovasc Res.

[124] Moreno A (2021). Optogenetic control of cardiac autonomic neurons in transgenic mice. Methods Mol Biol.

[125] Burton RB (2020). Optical interrogation of sympathetic neuronal effects on macroscopic cardiomyocyte network dynamics. iScience.

[126] Yu L (2017). Optogenetic modulation of cardiac sympathetic nerve activity to prevent ventricular arrhythmias. J Am Coll Cardiol.

[127] Nussinovitch U, Shinnawi R, Gepstein L (2014). Modulation of cardiac tissue electrophysiological properties with light-sensitive proteins. Cardiovasc Res.

[128] Quinn TA (2016). Electrotonic coupling of excitable and nonexcitable cells in the heart revealed by optogenetics. Proc Natl Acad Sci U S A.

[129] Fernandez MC (2021). Channelrhodopsins for cell-type specific illumination of cardiac electrophysiology. Methods Mol Biol.

[130] Funken M, Bruegmann T, Sasse P (2020). Selective optogenetic stimulation of fibroblasts enables quantification of hetero-cellular coupling to cardiomyocytes in a three-dimensional model of heart tissue. Europace.

[131] Wang Y (2023). Fibroblasts in heart scar tissue directly regulate cardiac excitability and arrhythmogenesis. Science.

[132] Yu J, Entcheva E (2016). Inscribing optical excitability to non-excitable cardiac cells: viral delivery of optogenetic tools in primary cardiac fibroblasts. Methods Mol Biol.

[133] Hulsmans M (2017). Macrophages facilitate electrical conduction in the heart. Cell.

[134] Entcheva E, Kay MW (2021). Cardiac optogenetics: a decade of enlightenment. Nat Rev Cardiol.

[135] Ambrosi CM (2014). Cardiac applications of optogenetics. Prog Biophys Mol Biol.

[136] Karathanos TV, Boyle PM, Trayanova NA (2016). Light-based approaches to cardiac arrhythmia research: from basic science to translational applications. Clin Med Insights Cardiol.

[137] Entcheva E, Bub G (2016). All-optical control of cardiac excitation: combined high-resolution optogenetic actuation and optical mapping. J Physiol.

[138] Richter C (2016). Optogenetic light crafting tools for the control of cardiac arrhythmias. Methods Mol Biol.

[139] Gepstein L, Gruber A (2017). Optogenetic neuromodulation of the heart. J Am Coll Cardiol.

[140] Koopman CD (2017). Cardiac optogenetics: using light to monitor cardiac physiology. Basic Res Cardiol.

[141] Ferenczi EA, Tan X, Huang CL (2019). Principles of optogenetic methods and their application to cardiac experimental systems. Front Physiol.

[142] Zgierski-Johnston CM, Schneider-Warme F (2021). Observing and manipulating cell-specific cardiac function with light. Adv Exp Med Biol.

[143] Tkatch T (2017). Optogenetic control of mitochondrial metabolism and Ca(2+) signaling by mitochondria-targeted opsins. Proc Natl Acad Sci U S A.

[144] Milligan G, Kostenis E (2006). Heterotrimeric G-proteins: a short history. Br J Pharmacol.

[145] Neves SR, Ram PT, Iyengar R (2002). G protein pathways. Science.

[146] Banghart M (2004). Light-activated ion channels for remote control of neuronal firing. Nat Neurosci.

[147] Volgraf M (2006). Allosteric control of an ionotropic glutamate receptor with an optical switch. Nat Chem Biol.

[148] van Wyk M (2015). Restoring the ON switch in blind retinas: Opto-mGluR6, a next-generation, cell-tailored optogenetic tool. PLoS Biol.

[149] Pu WT, Ma Q, Izumo S (2003). NFAT transcription factors are critical survival factors that inhibit cardiomyocyte apoptosis during phenylephrine stimulation in vitro. Circ Res.

[150] Lin TY et al (2023) Cardiac contraction and relaxation are regulated by distinct subcellular cAMP pools. Nat Chem Biol. 10.1038/s41589-023-01381-8 10.1038/s41589-023-01381-8 PMC10746541 37474759

[151] Ryu MH (2014). Engineering adenylate cyclases regulated by near-infrared window light. Proc Natl Acad Sci U S A.

[152] Ryu MH (2010). Natural and engineered photoactivated nucleotidyl cyclases for optogenetic applications. J Biol Chem.

[153] Etzl S (2018). Structure-guided design and functional characterization of an artificial red light-regulated guanylate/adenylate cyclase for optogenetic applications. J Biol Chem.

[154] Mukherjee S, Hegemann P, Broser M (2019). Enzymerhodopsins: novel photoregulated catalysts for optogenetics. Curr Opin Struct Biol.

[155] Hagio H et al (2023) Optogenetic manipulation of neuronal and cardiomyocyte functions in zebrafish using microbial rhodopsins and adenylyl cyclases. Elife 12:e83975 10.7554/eLife.83975 PMC10435232 37589546

[156] Gasser C (2014). Engineering of a red-light-activated human cAMP/cGMP-specific phosphodiesterase. Proc Natl Acad Sci U S A.

[157] Fukuda N, Matsuda T, Nagai T (2014). Optical control of the Ca2+ concentration in a live specimen with a genetically encoded Ca2+-releasing molecular tool. ACS Chem Biol.

[158] He L et al (2015) Near-infrared photoactivatable control of Ca(2+) signaling and optogenetic immunomodulation. Elife 4:e10024 10.7554/eLife.10024 PMC4737651 26646180

[159] Kyung T (2015). Optogenetic control of endogenous Ca(2+) channels in vivo. Nat Biotechnol.

[160] Kim JM (2005). Light-driven activation of beta 2-adrenergic receptor signaling by a chimeric rhodopsin containing the beta 2-adrenergic receptor cytoplasmic loops. Biochemistry.

[161] Tichy AM et al (2022) Structure-guided optimization of light-activated chimeric G-protein-coupled receptors. Structure 30(8):1075–1087 10.1016/j.str.2022.04.012 35588733

[162] Leemann S, Kleinlogel S (2023). Functional optimization of light-activatable Opto-GPCRs: illuminating the importance of the proximal C-terminus in G-protein specificity. Front Cell Dev Biol.

[163] Niu X (2012). Cardioprotective effect of beta-3 adrenergic receptor agonism: role of neuronal nitric oxide synthase. J Am Coll Cardiol.

[164] Salie R (2019). Cardioprotective effects of beta3-adrenergic receptor (beta3-AR) pre-, per-, and post-treatment in ischemia-reperfusion. Cardiovasc Drugs Ther.

[165] Takenouchi O, Yoshimura H, Ozawa T (2018). Unique roles of beta-arrestin in GPCR trafficking revealed by photoinducible dimerizers. Sci Rep.

[166] Kennedy MJ (2010). Rapid blue-light-mediated induction of protein interactions in living cells. Nat Methods.

[167] Hannanta-Anan P, Chow BY (2018). Optogenetic inhibition of Galpha(q) protein signaling reduces calcium oscillation stochasticity. ACS Synth Biol.

[168] O'Neill PR, Gautam N (2014). Subcellular optogenetic inhibition of G proteins generates signaling gradients and cell migration. Mol Biol Cell.

[169] Melero-Fernandez de Mera RM (2017). A simple optogenetic MAPK inhibitor design reveals resonance between transcription-regulating circuitry and temporally-encoded inputs. Nat Commun.

[170] Yi JJ (2014). Manipulation of endogenous kinase activity in living cells using photoswitchable inhibitory peptides. ACS Synth Biol.

[171] Maltan L et al (2021) Deciphering molecular mechanisms and intervening in physiological and pathophysiological processes of Ca(2+) signaling mechanisms using optogenetic tools. Cells 10(12):3340 10.3390/cells10123340 PMC8699489 34943850

[172] Ma G (2020). Optogenetic engineering to probe the molecular choreography of STIM1-mediated cell signaling. Nat Commun.

[173] Kim S (2020). Non-invasive optical control of endogenous Ca(2+) channels in awake mice. Nat Commun.

[174] Ishii T (2015). Light generation of intracellular Ca(2+) signals by a genetically encoded protein BACCS. Nat Commun.

[175] Bohineust A (2020). Optogenetic manipulation of calcium signals in single T cells in vivo. Nat Commun.

[176] He L (2021). Engineering of a bona fide light-operated calcium channel. Nat Commun.

[177] Murakoshi H (2017). Kinetics of endogenous CaMKII required for synaptic plasticity revealed by optogenetic kinase inhibitor. Neuron.

[178] Shen Y (2020). Challenges for therapeutic applications of opsin-based optogenetic tools in humans. Front Neural Circuits.

